# Long-term survival of interval breast cancers in breast cancer screening in Wales

**DOI:** 10.1186/bcr3254

**Published:** 2012-11-09

**Authors:** YF Fong, J Evans, D Brookes, K Gower Thomas

**Affiliations:** 1 Royal Glamorgan Hospital, Llantrisant, UK; 2 Breast Test Wales, Cardiff, UK

## Introduction

Breast Test Wales is part of the NHS Breast Cancer Screening Programme, and oversees the screening programme in Wales. It is successful in identifying asymptomatic cancers; however, interval cancers (IC) still occur between screenings. We aim to evaluate the overall long-term survival of IC and compare that with screen-detected cancers (SDC).

## Methods

Within BTW, SDC between 1998 and 2001 and IC occurring between 1998 and 2004 but screened between 1998 and 2001 were identified. IC were classified into true interval (TI), false negative (FN), occult (OCC) and unclassified (UCC). BTW receives notification of death of all women that underwent screening. The long-term survival rate was calculated from the date of initial screening and the date of death.

## Results

In the 3-year screening period, 199,082 women were screened. A total of 1,020 women had SDC and 692 further developed IC following screening. Of the 692 IC, 57.8% (391) were TI, 17.7% (120) were FN, 10% (68) were OCC and 14.5% were UCC. After at least 10 years of follow-up, the long-term survival rate (all-cause) for SDC was 81.6%, overall for IC was 72.4% (OR 1.67, *P *< 0.001), TI was 77.5% (OR 1.00, *P *= 0.99), FN was 55% (OR 2.36, *P *< 0.001), OCC was 54.4% (OR 3.17, *P *< 0.001) and UCC was 87.8% (OR 0.61, *P *= 0.19).

## Conclusion

Overall survival of IC is significantly different to SDC. However, SDC and TI were not statistically significantly different. FN and OCC had significantly worse long-term survival. Further research is required to identify the underlying cause of poor survival of FN and OCC.

